# Management of Acute Coronary Syndrome Following Blunt Chest Trauma: A Case Report

**DOI:** 10.30476/BEAT.2021.87689.1192

**Published:** 2021-07

**Authors:** Ee Lyn Chan, Jawaad Saleem Malik, Carlos Gomez

**Affiliations:** 1 *Junior Clinical Fellow in Intensive Care Medicine, St Mary’s Hospital, Imperial College NHS Trust, London, United Kingdom*; 2 *Anaesthetic Registrar, St Mary’s Hospital, Imperial College NHS Trust, London, United Kingdom *; 3 *Consultant and Honorary Senior Lecturer in Intensive Care Medicine and Anaesthesia, St Mary’s Hospital, Imperial College NHS Trust, London, United Kingdom*

**Keywords:** Acute myocardial infarction, Trauma, Angiogram, Emergency medicine, Intensive care

## Abstract

Blunt chest trauma is a rare cause of acute coronary syndrome and can be masked by other injuries in polytrauma patients. It can have devastating consequences due to damage to the myocardial tissue if left un-recognized. Myocardial injury can result in life-threatening arrhythmias and complications such as systolic and diastolic dysfunction. This can significantly affect patients’ quality of life. A 34-year-old man involved in a paragliding incident in Kazakhstan. His equipment failed at 30 meters height and result him to be propelled at high velocity to the ground. He sustained multiple injuries including spinal fractures, lung contusions and a mediastinal haematoma. He was transported to a local hospital and noted to have ST segment elevation on his admission electrocardiogram (ECG). He underwent an angiogram that showed sub-occlusion of his left anterior descending (LAD) artery. This resulted in a time-critical Percutaneous Coronary Intervention (PCI). He was stabilized and repatriated to the UK to manage of remaining injuries.

## Introduction

Non –penetrating trauma to the heart and complications are relatively rare. However, consequences of cardiovascular injury such as myocardial rupture of a septum or chamber wall and thrombosis of major coronary artery are significant therefore they should be assessed and managed appropriately [1]. The use of PCI for timely restoration of coronary perfusion is critical if coronary artery injury is present and is the preferred intervention in polytrauma patients with high risk of bleeding. These cohorts of patients are likely to have other distracting injuries that need to be considered during management and should be approached in a multi-disciplinary manner with input from other specialist teams.

## Case Presentation

**Fig. 1:**
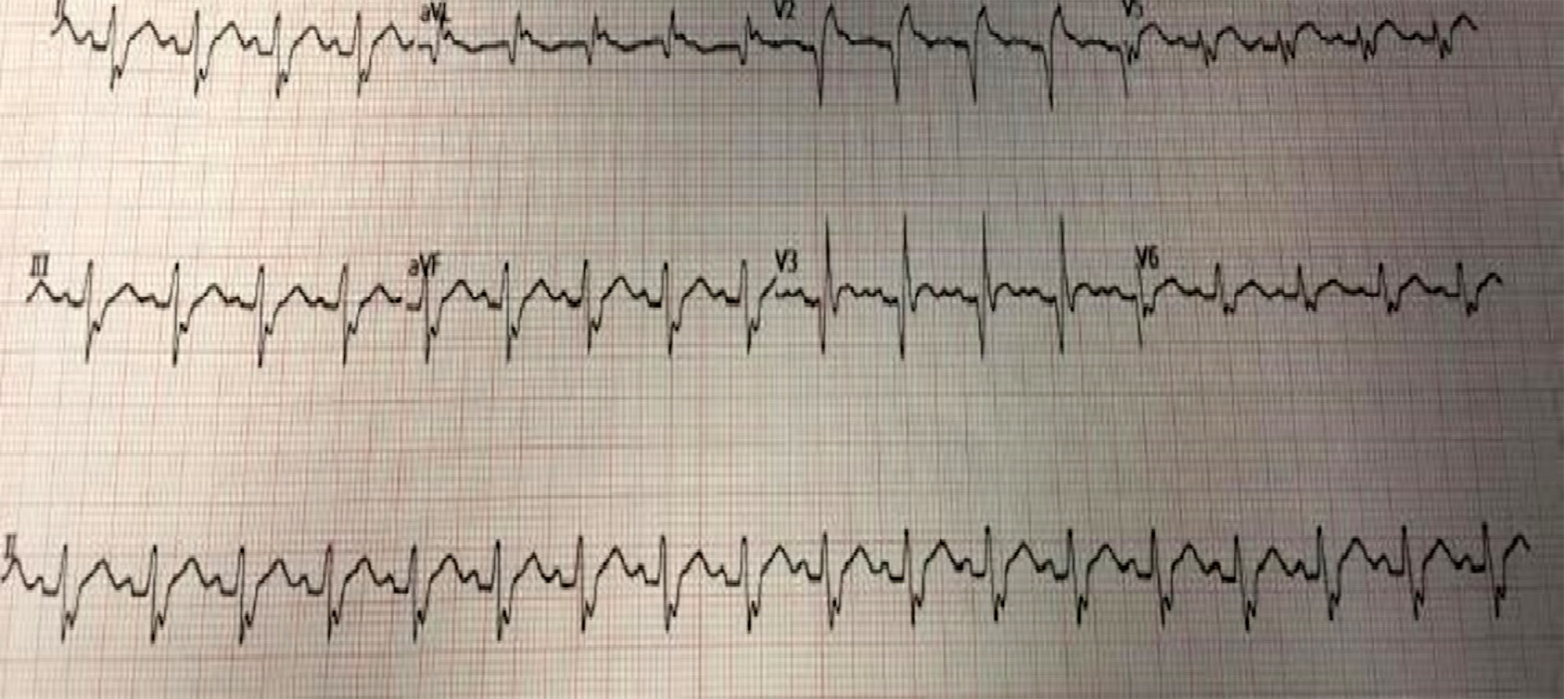
ECG after repatriation

**Fig. 2:**
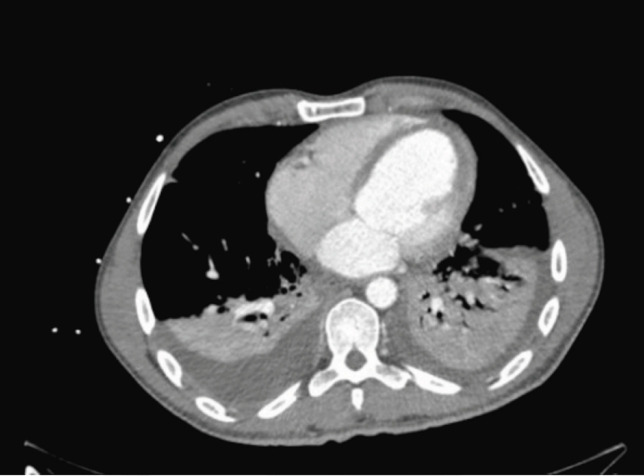
Anterior mediastinal haematoma on CT scan

A 34-year-old gentleman who was previously fit and well was admitted to a hospital in Kazakhstan following a paragliding accident. He fell onto the ground from approximately 30 meters during his descent. On admission to the emergency department in Kazakhstan, he was found to have ST elevation in serial ECGs and a raised troponin of 83246 µg/L (Figure 1). A pan CT revealed multiple injuries including C7 anterior vertebral body fracture, thoracic and lumbar spine fractures, bilateral haemothoraces, pulmonary contusions, mediastinal haematoma, adrenal haematoma, splenic laceration and renal infarcts (Figure 2). 

**Fig. 3:**
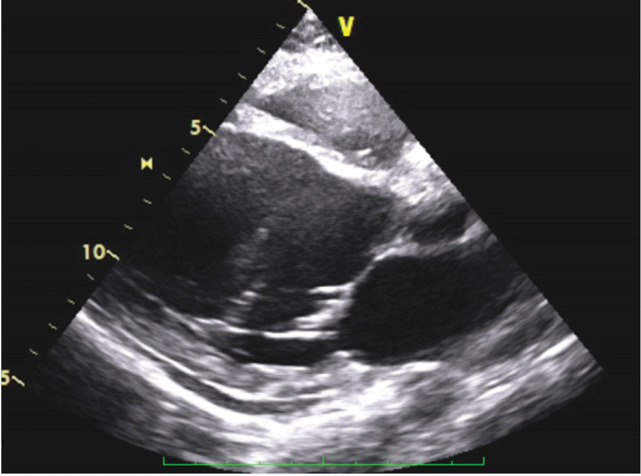
Echocardiogram post PCI

**Fig. 4:**
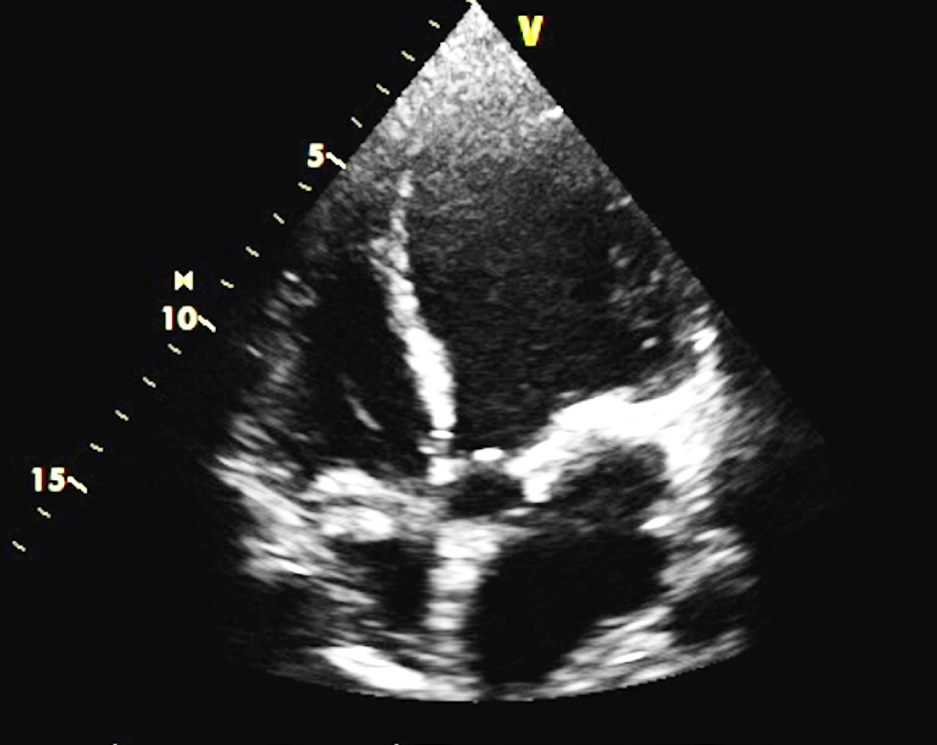
Echocardiogram post PCI

He underwent an angiogram which showed sub-occlusion of left anterior descending artery (LAD). Subsequently, he had a PCI to the LAD with a drug eluting stent (DES- Resolute Onyx) and was started on dual anti-platelet therapy. Then, he was repatriated back to the United Kingdom where he received care in a tertiary trauma center. During this period, he developed a hospital-acquired pneumonia and was admitted to the intensive care for humidified high flow oxygen therapy and antibiotics. Echocardiography revealed that patient had a severely impaired left ventricular ejection fraction of between 25-30% (Figure 3 and 4) upon repatriation to our unit. Poor ejection fraction could be multifactorial taking into account possibility of cardiac contusion from injury as well as pulmonary contusion [2, 3].

He was started on a B blocker and Angiotensin-converting-enzyme (ACE) inhibitor to manage his heart failure. Subsequent investigations revealed improvement of ejection fraction to above 60%. A multi-disciplinary meeting concluded that the perioperative risk for fixation of his spinal fracture was too high this soon after his injury and that he should continue on dual antiplatelets for 4 weeks prior to an operation. The patient’s C-spine injury was managed conservatively in a Miami J Collar during this period, initially as an inpatient, then at home. 

Unfortunately, he developed a subluxation of C6/C7 fracture with associated C7 collapse a shortly after discharge on a repeat CT scan. Despite high cardiac and bleeding risks, the patient underwent a C7 corpectomy and spinal fixation after multidisciplinary discussion between teams.

Outcome and Follow-Up

Patient had anterior corpectomy of C7 and spinal fixation a month after his initial injury whilst on dual antiplatelet therapy. He was admitted to ICU post operatively for monitoring and blood pressure control and was subsequently discharged three days later with no neurological sequelae. He was discharged on heart failure medications with a follow up appointment with the heart failure team. 

## Discussion

Blunt trauma to chest wall can cause of myriad of complications [1]. Coronary occlusion is a rare occurrence in these patients and can be caused by a variety of aetiologies including coronary artery rupture and dissection, embolism of pre-existing atherosclerotic plaque, external compression and vascular spasm at the site of the injury. Unfortunately, we were unable to obtain images from the coronary angiogram and angioplasty images as the procedure was done in Kazakhstan and thus, unable to ascertain exact aetiology of sub-occlusion. However, it is of importance to detect and manage these aetiologies to avoid possible long-term consequences as listed below. 

There have been multiple reports of ST-elevation myocardial infarction (STEMI) post non- penetrating trauma of which some were treated conservatively or with thrombolysis [4-6]. In addition to that, on pump and off pump coronary artery bypass grafting (CABG) has been used as a successful treatment modality in some instances [7-9]. However, it can be associated with complications including bleeding due to systemic anticoagulation as well as possible pulmonary, renal and neurological side effects [10]. Therefore, choice of treatment should be considered on a case-to-case basis in polytrauma patients - taking into account other injuries, anatomical cardiac lesion and patients’ pre-morbid status. 

Focusing on the use of PCI as a treatment option, cases have demonstrated importance in the role of PCI to manage these patients with definitive occlusion on angiogram in order to prevent delayed management and subsequent consequences such as congestive heart failure [11-13]. PCI has been shown to have higher rates of reperfusion and lower risk of re-occlusion and re-infarction [13]. However, the use of high dose anti-platelet therapy prior to PCI and the need for maintenance of these medications need to be taken into consideration as this might pre-dispose patients to worsening of existing injuries and complicate planning for further operations. 

Patients with traumatic injuries who undergo PCI with drug-eluting stent (DES) are usually started on dual-antiplatelets for 6 to 12 months [14]. This is to optimize reduction in risk of stent thrombosis and minimize risk of bleeding [14 ,15]. Our patient’s spinal fracture was initially managed conservatively due to the high risk secondary to his recent STEMI and high bleeding risk as he was on dual antiplatelet therapy. However, due to the progressive worsening of spinal fracture and increased risk of compression to his spinal cord, decision was made to proceed with surgery despite a risk of a major adverse cardiac event and bleeding secondary to dual antiplatelet therapy. The complex clinical challenge here was to strike a balance between spinal surgery to prevent life changing neuro-disability versus allowing for restoration of coronary anatomy and flow which required multi-disciplinary input.

## Conclusion

In conclusion, myocardial infarction post blunt trauma is a rare complication however has catastrophic implications and should be recognized as well as managed in a time critical manner. Patients with polytrauma will present with other debilitating injuries, these should be addressed with a holistic and multi-disciplinary approach to ensure optimal patient care is achieved.

Take Home Messages 

Main causes of ST elevation MI post blunt trauma include coronary artery dissection, thrombosis of vessel from pre-existing atherosclerotic disease

Coronary angiography is a good tool in diagnosing coronary vessel injury; other investigations such as serial ECGs, troponin and trans-oesophageal echocardiography can give indications of the injury. 

Optimal treatment of STEMI in blunt trauma is still debatable however there is a growing role of PCI in these patients 

Duration of dual anti-platelet therapy should ideally be between 6-12 months however should be assessed on a case to case basis in a multi- disciplinary setting involving trauma surgeons, anaesthetists, cardiologists and intensivists.

## Conflict of Interests:

None declared.
